# EXPERIENCES RESEARCH ON CANCER-RELATED FATIGUE AMONG OLDER ADULTS

**DOI:** 10.1093/geroni/igz038.2376

**Published:** 2019-11-08

**Authors:** Susan M Hannum, Katherine Smith, Dori M Beeler, Jill Owczarzak

**Affiliations:** 1 Johns Hopkins Bloomberg School of Public Health, Baltimore, Maryland, United States; 2 Johns Hopkins School of Public Health, Baltimore, Maryland, United States

## Abstract

Among older cancer patients, cancer-related fatigue is a common chronic problem that may result in functional dependence and decreased quality of life. Currently, there exists no standard of care for managing fatigue among cancer patients, pressing the need for further research and the development of novel interventions. Our research followed the validated DIPEx (Database of Patient Experiences) methodology, which utilizes in-depth, narrative interviews to create a rich and broad archival resource of patient stories to highlight commonalities and differences in illness experiences. We interviewed 9 older women (aged 60+ years) who have been diagnosed with and treated for breast cancer (Stage I-IV (IV only if clinically-stable). Interviews were audio and/or video-recorded and transcribed for analysis of emergent themes using MAXQDA qualitative software. Four primary themes have emerged from the data: (1) fatigue is a distressing side effect of treatment for which patients do not feel adequately prepared; (2) information about fatigue and how to deal with it is not systematically provided within the oncology setting; (3) patients develop their own systems for managing fatigue and general energy levels (e.g., limiting activities, using blocks of time strategically, etc.); and (4) social support for fatigue varies. Education about cancer-related fatigue and its management represents an unmet need among older breast cancer survivors. The development and implementation of both clinician training initiatives and patient-facing educational and engagement interventions represent important next steps in supporting the care needs of cancer patients and survivors.

